# A disease resistance locus on potato and tomato chromosome 4 exhibits a conserved multipartite structure displaying different rates of evolution in different lineages

**DOI:** 10.1186/s12870-015-0645-8

**Published:** 2015-10-24

**Authors:** Marialaura Destefanis, Istvan Nagy, Brian Rigney, Glenn J Bryan, Karen McLean, Ingo Hein, Denis Griffin, Dan Milbourne

**Affiliations:** Crops, Environment and Land Use Programme, Teagasc, Oak Park, Carlow Ireland; Cell and Molecular Sciences, The James Hutton Institute, Dundee, DD2 5DA UK; Pesticides, Plant Health & Seed Testing Laboratories, Department of Agriculture, Food and the Marine, Backweston Campus, Celbridge, Co. Kildare Ireland; Department of Molecular Biology and Genetics, Aarhus University, Forsøgsvej 1, 4200 Slagelse, Denmark

## Abstract

**Background:**

In plant genomes, NB-LRR based resistance (R) genes tend to occur in clusters of variable size in a relatively small number of genomic regions. R-gene sequences mostly differentiate by accumulating point mutations and gene conversion events. Potato and tomato chromosome 4 harbours a syntenic R-gene locus (known as the *R2* locus in potato) that has mainly been examined in central American/Mexican wild potato species on the basis of its contribution to resistance to late blight, caused by the oomycete pathogen *Phytophthora infestans*. Evidence to date indicates the occurrence of a fast evolutionary mode characterized by gene conversion events at the locus in these genotypes.

**Results:**

A physical map of the *R2* locus was developed for three *Solanum tuberosum* genotypes and used to identify the tomato syntenic sequence. Functional annotation of the locus revealed the presence of numerous resistance gene homologs (*RGHs*) belonging to the *R2* gene family (*R2GH*s) organized into a total of 4 discrete physical clusters, three of which were conserved across *S. tuberosum* and tomato. Phylogenetic analysis showed clear orthology/paralogy relationships between *S. tuberosum R2GH*s but not in *R2GH*s cloned from *Solanum* wild species. This study confirmed that, in contrast to the wild species *R2GH*s, which have evolved through extensive sequence exchanges between paralogs, gene conversion was not a major force for differentiation in *S. tuberosum R2GH*s, and orthology/paralogy relationships have been maintained via a slow accumulation of point mutations in these genotypes.

**Conclusions:**

*S. tuberosum* and *Solanum lycopersicum R2GH*s evolved mostly through duplication and deletion events, followed by gradual accumulation of mutations. Conversely, widespread gene conversion is the major evolutionary force that has shaped the locus in Mexican wild potato species. We conclude that different selective forces shaped the evolution of the *R2* locus in these lineages and that co-evolution with a pathogen steered selection on different evolutionary paths.

**Electronic supplementary material:**

The online version of this article (doi:10.1186/s12870-015-0645-8) contains supplementary material, which is available to authorized users.

## Background

Plants have evolved sophisticated mechanisms to defend themselves from attack from biotic threats. Intracellular defence against pathogens and pests is locally and systematically promoted by mechanisms of incompatible interaction, which are coordinated by resistance (R) genes [1]. The largest class of plant R-genes encodes for modular proteins characterized by a nucleotide binding (NB) site and leucine-rich repeat (LRR) domains. The NB domain regulates the downstream signalling of the defence response to pathogens [2, 3]; while recognition of non-self is granted by the LRR domains located at the C terminus of NB-LRR proteins [3–5].

In plant genomes, R-genes tend to occur as tightly linked gene clusters, where duplication is achieved via illegitimate recombinational processes, comprising groups of closely related R-gene sequences or R-gene homologs (*RGH*s) [6–8]. This organization in tandem arrays of R-genes provides a broader potential range of responses to multiple pathogens and to different variants of the same pathogen [9].

The tendency of R-genes to cluster in plant genomes facilitates sequence exchanges that enable rapid rearrangements necessary to respond to changes in the pathogen population. Despite this, Song et al. [9] showed that *RGH*s at the *RB* locus in potato evolve independently and mostly through point mutations rather than sequence exchanges. This sort of evolution contributes to the maintenance of orthologous relationships between homologs in different genotypes, while the frequent sequence exchanges and gene conversion events act to obscure this association [10]. Based on these observations, Kuang et al. [10] hypothesized that plant disease R-genes are organized in two classes that explain the evolutionary patterns with contrasting rates of evolution. Type I R-genes evolve rapidly through frequent sequence exchanges, while Type II R-genes evolve slowly and in an autonomous manner. The two gene types can be present at the same cluster or individually, and they can have different frequencies in natural populations and within species [10, 11].

The tuber crop potato (*Solanum tuberosum*) is ranked as the world’s third most important food crop and a prominent member of the Solanaceae, a botanical family including the closely related model species tomato. Potato is susceptible to many pests and diseases, which can affect all parts of the plant and cause severe reduction both in tuber quality and quantity. The number of sequence-based comparative analyses of potato R-gene loci has recently increased [12–16], due to some degree to the recent publication of the potato genome sequence [17]. R-genes in solanaceous genomes tend to be distributed in restricted chromosome regions that are conserved within the members of the family [7, 8, 18]. However, syntenic genes in *Solanum* R-gene clusters acquired different specificities owing to the interaction with different pathogens [7, 8, 19]. Potato and tomato chromosome 4 harbour a syntenic complex R-gene region. In potato, this region is a hotspot for resistance, which exhibits both qualitative and quantitative resistance to different pathogens and pests [20]. No contribution to functional resistance has yet been detected at the locus in tomato. The oomycete *Phytophthora infestans* (causal agent of late blight of potatoes) is considered the dominant biotic stress afflicting potato worldwide, not only because of its capability to rapidly destroy untreated potato crops but also for itspotential to repeatedly adapt to resistant varieties in only a few years [21]. Numerous race-specific late blight R-genes from the resistance hotspot on chromosome 4 have been recently cloned from different *Solanum* wild species: *R2* (from *Solanum demissum*), *R2-like, Rpi-edn1.1* (from *Solanum edinense*), *Rpi-abpt* [from *Solanum* ABPT (derived from inter-specific bridge crosses between *Solanum bulbocastanum*, *Solanum acaule*, S*olanum phureja*, and *S. tuberosum* [22])], *Rpi-blb3* (from *S. bulbocastanum*), *Rpi-hjt1.1, Rpi-hjt1.2, Rpi-hjt1.3* (from *Solanum hjertingii*), *Rpi-snk1.1* and *Rpi-snk1.2* (from *Solanum schenckii*) [23, 24]. All genes encode for NB-LRR proteins approximately 846 amino acids long. They belong to the same family as they share very high nucleotide identity level (from 95 to 99 %) and their products are known to interact with four members of the PiAvr2 family of blight effectors [25]. Quantitative resistance loci (QRLs) for both blight and potato cyst nematode have also been mapped to this region [20], with the resistance phenotypes generally being ascribed to the presence of R-genes. Because the late blight R-gene *R2* from *S. demissum* was the first to be mapped to this locus [26], for brevity this chromosome 4 hotspot for resistance and all R-gene homologs will hereafter be referred as *R2GH*s from the *R2* region.

The *R2GH*s cloned at the *R2* region in *Solanum* wild species (*S. demissum, S. bulbocastanum*, *S. edinense*, *S. schenckii*, *S. hjertingii)* and the breeding clone *Solanum* ABPT have been described as “patchworks of sequence similarity” [23, 24]; suggesting that they can be classified as Type I R-genes. This picture, however, has emerged from a very specific focus on wild Mexican species originating from the centre of diversity of the late blight pathogen. Based on the availability of the genome sequence of potato, we have tried to gain insights into the structure and evolution of the *R2* region in genotypes of the South American species *S. tuberosum* and the closely related species tomato. The analysis of the region was carried out in the three *S. tuberosum* genotypes, DM1-3 516R44 (DM), RH89-039-16 (RH) (respectively, the doubled monoploid and diploid heterozygous clones recently used for the draft sequence of the potato genome) and the heterozygous diploid genotype HB171(13) (HB), and involved comparisons to the whole genome sequence of tomato generated for the cultivar Heinz 1706. The region is revealed to be characterized by a multipartite clustered organization and to show a high level of colinearity in the genotypes analyzed. In contrast to the genes isolated from wild species to date, it seems that the *R2GH*s in the genotypes investigated in this study evolved slowly and through the accumulation of point mutations, implying that two different patterns of evolution characterize members of the *R2* region in different *Solanum* species.

## Results

### Organization and structure of the *R2* gene family in potato and tomato genotypes

A total of 9 bacterial artificial chromosome (BAC) clones spanning ~780 Kbp of the *R2* locus were sequenced in one haplotype (RH-H0) of the diploid potato clone RH. Lower sequence coverage was achieved for the HB diploid clone, where ~430 Kbp were sequenced in the HB-H0 haplotype and ~122 Kbp in the HB-H1 haplotype, which is known to carry alleles for quantitative resistance to blight derived from an introgression from *S. demissum* [27]. An approximately 3 Mb region of the current pseudomolecule build of DM [28] spanning the *R2* region was identified in the DM genome. This region comprises 7 ‘superscaffolds’ , 6 of which contain R2GHs, with a single interstitial superscaffold not containing R2GHs. Close examination of the DM sequence in the current pseudomolecule build, and comparison to tomato and the other potato genotypes, suggested that three contiguous scaffolds in the region (PGSC0003DMB000000690, PGSC0003DMB000000707 and PGSC0003DMB000000964) have been placed in the incorrect orientation. Two further superscaffolds containing R2GHs (PGSC0003DMB000000719 and PGSC0003DMB000001035) cannot be placed in the region with high confidence following reorientation of the three inverted scaffolds. The revised order actually corresponds to the organisation of these scaffolds in builds of the potato genome prior to the current pseudomolecule assembly, where PGSC0003DMB000000690, PGSC0003DMB000000707 and PGSC0003DMB000000964 were in the opposite orientation, and PGSC0003DMB000000719 and PGSC0003DMB000001035 were unanchored. For the purpose of this study, we have adopted this previous, in our view, more likely arrangement at the R2 locus for DM. A single scaffold of 11.6 Mbp (SL2.40sc03604) syntenic to the *R2* region in potato was identified from the tomato cultivar Heinz 1703 (HZ) genome sequence (Tomato WGS scaffolds, SC2.40), which formed the basis of the potato/tomato comparisons. Additional file 1 shows the complete sequence coverage of the region in different potato genotypes and tomato, with a revised version of the DM region assembly comprising 5 superscaffolds that can be placed in the region with high confidence.

Functional annotation of the region in the four genotypes investigated revealed a complex structure including several apparently discrete sub-clusters of *R2* R-gene homologues intermingled with relatively large inter-cluster regions. Each inter-cluster region harbours a minimum of eight open reading frames not directly associable to disease resistance, which, according to Richly et al. [29], allows definition of each of the R-gene-containing regions as independent *RGH* clusters. DM, the potato genotype with the greatest coverage, possesses four discrete clusters spread across ~1500kbp. RH, in which there is near complete sequence coverage in a span of ~780kbp in a single haplotype, exhibits three clusters. Preliminary examination of the flanking regions surrounding the *R2GH* clusters in RH indicated that they occupy syntenic positions to three of the DM clusters, with the position of the fourth DM cluster falling outside the window of coverage in RH. Even though the sequence coverage in both HB haplotypes is incomplete, the three clusters covered in RH were also covered in this genotype, with no sequence coverage in the region of the fourth DM cluster. (Additional file 1 gives a comprehensive diagrammatic representation of region in all sequenced genotypes). Tomato also exhibited three discrete clusters that seemed to occupy syntenic positions to the three clusters covered across all of the potato genotypes. Tomato does not possess an equivalent cluster in the region of the fourth cluster identified only in DM, despite good coverage in this region of the tomato assembly.

We compared the region, spanning the three apparently syntenic R-gene clusters, with equivalent sequence coverage across three potato genotypes and tomato. This region in the potato genotypes DM and RH-H0 spans approximately 800 Kbp, whereas the tomato equivalent region covers a smaller area of ~550 Kbp. In both species the region includes a distal R-gene cluster (A), a central cluster (B) and a proximal cluster (C). Orientation of transcription and number of R-gene homologues at each cluster are generally well conserved. All *R2GHs* in cluster B and C possess the same orientation, whereas cluster A members are in inverse orientation relative to these clusters in both species. In all genotypes, Cluster A harbours the highest number of *R2GH*s, followed by cluster B, with Cluster C containing the fewest. While Clusters B and C are of similar size across potato and tomato, potato genotypes possess approximately three times as many R2GHs as tomato at Cluster A (Additional file 1). RH, DM and HB(H0) share highly collinear and nearly completely conserved micro-syntenic inter-R gene-cluster sequences (Additional file 2). Potato and tomato also show extensive colinearity in the inter-R gene-cluster sequences. Five potato-tomato microsyntenic blocks including up to 14 pairs of homologous genes within comparable physical distance were identified. Microsynteny between the two species is only interrupted by unilateral insertions of transposable elements in the potato sequences (Additional file 3).

The fourth cluster, found only in DM, is approximately 370 kb further proximal to cluster A and harbours 2 *R2GH*s (see Additional file 1). Sequence coverage did not extend to this region in potato genotypes RH and HB, and a syntenic cluster may or may not exist in these genotypes. As mentioned, no further R2GHs were found in the equivalent region proximal to cluster A in tomato despite adequate sequence coverage.

The availability of the entire genomic sequence in DM and HZ, allowed the identification of further *R2* resistance gene homologs located outside the *R2* region. These ectopic R-genes homologs can be considered members of the *R2* gene family, as they show >77 % identity at nucleotide level to other members at the *R2* region, although they were transferred to different genomic locations [30]. As previously mentioned, superscaffolds PGSC0003DMB000000719 and PGSC0003DMB000001035 are present in the *R2* region (between PGSC0003DMB000000964 and PGSC0003DMB000000355), in the current pseudochromosome molecule build of potato chromosome 4, and contain 4 and 2 R2GHs respectively. As described earlier, on the re-inversion of contiguous superscaffolds PGSC0003DMB000000690, PGSC0003DMB000000707 and PGSC0003DMB000000964, these can no longer be placed confidently into this region, and thus we treat them as unanchored for the purposes of this study.

Another superscaffold, PGSC0003DMB000000029, containing a single, *R2GH*, is present on potato chromosome 4, 15.6 Mbp from the *R2* region. In tomato three further ectopic *R2* homologs were identified on chromosome 4 (20 Mb proximal to the *R2* region) and chromosome 7.

### Sequence analysis of *R2GH*s in the *R2* region

Sequence annotation of potato and tomato genotypes revealed a total of 127 genes belonging to the *R2* gene family in the region being examined, 80 at cluster A, 33 at cluster B and 12 at cluster C and 2 at the additional cluster evident only in DM. (Additional file 4). To gain a better insight into *R2GHs* in the *R2* region of the genotypes examined, all R-genes were manually inspected and classified as full-length open reading frames or pseudogenes.

Of the 127 *R2GH*s 67 were classified as partial gene sequences either being truncated at the 5’ or 3’ termini, or containing internal deletions. The partial genes length varies from 345 bp (*RH0A012*) to 2529 bp (*RH0A007*). The remaining 58 full-length homologues are intron-less genes with an average length of 2530 (±41.7) bp and containing 3’ and 5’ UTRs of variable length. Of these full-length homologues, five contain premature stop codons and 15 exhibit frame shifts caused by nucleotide insertion or deletion. Thus, 87 sequences were classified as pseudogenes and 38 as full-length open reading frame genes. Full length ORF *R2GH*s encode for a peptide of ~843 aa, similar in structure to those described by Lokossou [24] and Champouret [23] for 40 members of the *R2* gene family cloned in *Solanum* wild species and cultivars [23, 24]. These include 31 full length ORF R-gene homologues (15 *RGH*s from *S. demissum*, nine *RGH*s from S*. edinense*, one *RGH* from *S. bulbocastanum*, one *RGH* from *S. hjertingii*, four *RGH*s from *S. schenckii* and one *RGH* from the interspecific cross ABPT, [22]) that are not involved in conferring resistance to blight; and nine functional genes *R2* (from *S. demissum*), *R2-like, Rpi-edn1.1* (from *S. edinense*), *Rpi-abpt* (from *Solanum* ABPT), *Rpi-blb3* (from *S. bulbocastanum*), *Rpi-hjt1.1, Rpi-hjt1.2, Rpi-hjt1.3* (from *S. hjertingii*), *Rpi-snk1.1* and *Rpi-snk1.2* (from *S. schenckii*) [23, 24]. All members of the *R2* gene family mapping to the *R2* locus were involved in further analysis of the mode of their evolution, including pseudogenes with a length greater than 1.5 Kbp.

### Establishing orthologous relationships between *R2GH*s

**Fig. 1:**
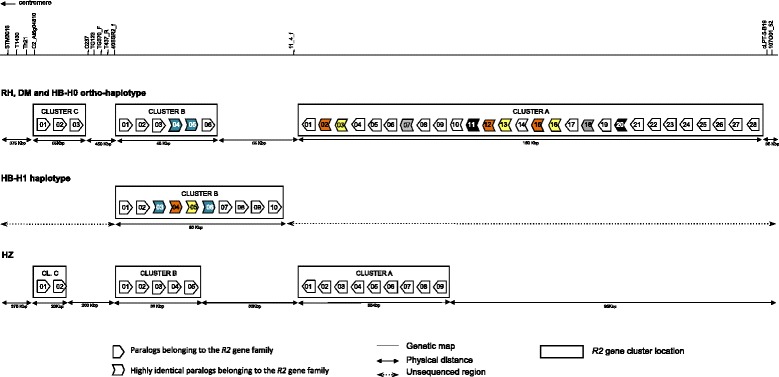
Physical and genetic location of R-gene clusters in the *R2* locus in the potato RH-H0, DM and HB-H0 ortho-haplopype, in the HB-H1 haplotype and in the tomato HZ genotype. The top of the figure represents the physical location of genetic markers used to map quantitative and qualitative resistance to pest and pathogens at the *R2* locus (STM3016 from Milbourne et al. .[42]; T1430, C2_At5g04810, TG123, TG370_F, T437_R, cLPT5-B19 from Bombarely et al. [43]; Th21 from Park et al. [44], C237 from Moloney et al. [39]; 11_4_f, 107O01_52, 40SSR2_f from Destefanis et al. unpublished results). The sequence of the *R2* region in RH, DM and HB-H0 is represented as part of the same ortho-haplotype because of the extensive conserved orthology amongst the three haplotypes. The sequence of HB-H1 structurally forms an independent haplotype from the ortho-haplotype. Only genes belonging to the *R2* gene family are represented as open arrows enclosed into boxes separating R-gene clusters. Conserved paralogs are highlighted in the same colours and patterns. The figure was not drawn in scale

The very similar numbers of *R2GH*s present at clusters A, B and C, across the potato genotypes examined (Additional files 1 and 4; Fig. 1) suggested that, contrary to what has been observed at most other complex plant R-gene loci [17, 29], a high degree of positional orthology exists for *R2GH*s at the locus in RH, DM, HB and to some extent also between potato and tomato.

**Fig. 2:**
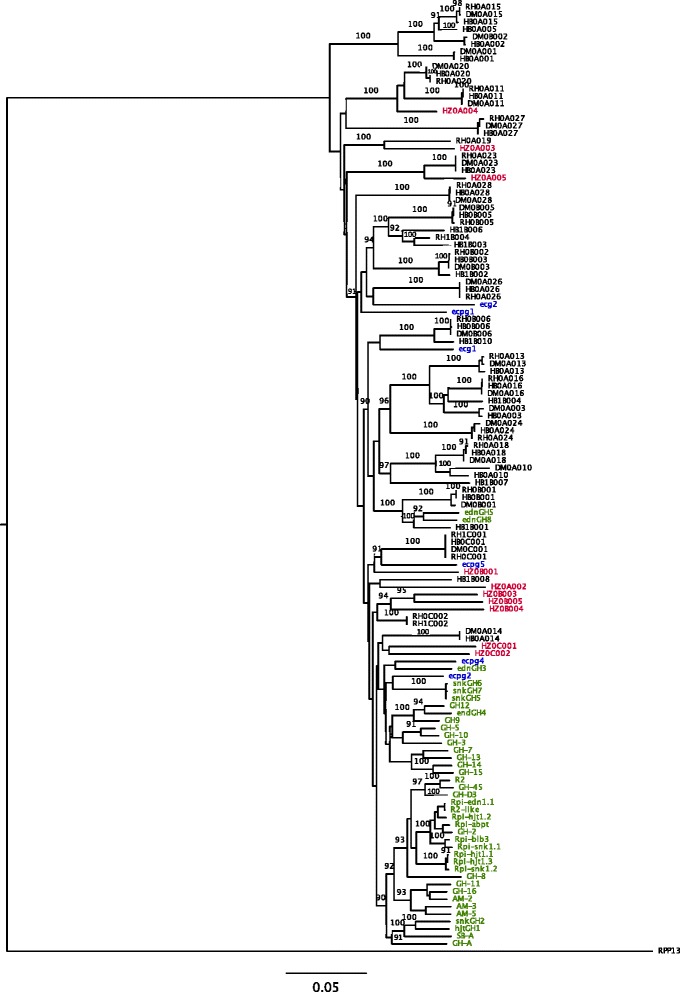
Dendrogram showing distance relationships among *R2* homologues. *R2GH*s from RH, DM and HB are shaded in black, whereas homologs from *Solanum* wild species and from tomato genotype HZ are shaded in green and red, respectively. Genes from domesticated and wild species do not generally mix and show different patterns of sequence diversity. The *Arabidopsis thaliana* gene *RPP13*, was used as an outgroup. The scale at the bottom is in units of nucleotide substitutions per site.

A multiple alignment for the *R2GH*s included in the analysis, using the closest non-Solanaceous R-gene homolog *RPP13* from *Arabidopsis thaliana* (accession FJ624096) as an outgroup, was used to generate a neighbour joining phylogenetic tree, which clearly shows the positional orthology between *R2GH*s occupying syntenic positions in different haplotypes (Fig. 2). Despite the lack of completely contiguous sequence coverage, the *R2GH*s identified in RH-H0, HB-H0 and DM exhibit apparent orthology with a “core” set of 28, 6 and 3 members at clusters A, B and C, respectively. Overall, across these three haplotypes there seems to exist a single “ortho-haplotype” comprising the same number of collinear *R2GH*s at the three clusters (Fig. 1). Despite their divergent recent origin - RH is a diploid potato breeding clone from the Netherlands, HB is a diploid clone derived from a cross between a primary dihaploid (PDH247) with *S. demissum* in its pedigree and a Group Phureja clone (DB226(70)) described in Bradshaw et al. 2006 [27], and DM is a doubled monoploid experimental clone [28] - all three genotypes have extensive contributions from *S. tuberosum* Group Phureja in their background (25 % in RH, 50 % in HB and 100 % in DM). Thus our reconstructed haplotypes (in RH, DM and HB-H0) are likely to mostly represent this lineage.

HB-H1, for which we have a limited picture, may reflect a combination of *S. tuberosum* and *S. demissum.* It’s dihploid parent PDH247 is similar in background to the cultivar Stirling, in which a large effect quantitative trait locus (QTL) for blight resistance was mapped in this region, and the same QTL has been mapped in HB171 [27, 31]. Given that the blight resistance in this background originates from *S. demissum*, then one might expect an introgression segment from that source in this region of HB-H1, although the boundary of that segment with the “receptor” *S. tuberosum* genome is unidentified. Despite the relatively low sequence coverage of this haplotype, it was apparent that cluster B had expanded relative to the other haplotypes (Fig. 1). Initial examination suggested that some form of sequence re-arrangement had occurred between clusters A and B (this is discussed further below).

The tomato *R2GH*s diverged to a greater extent from potato homologs, and, unsurpringly, the larger taxonomic gap meant that few orthologous relationships were apparent between members of equivalent clusters in potato and tomato. However, for three members of cluster A it was possible to identify orthologous relationships between potato and tomato.

*R2GH*s from *Solanum* wild species did not exhibit any clear orthologous relationships with the *S. tuberosum* lineage *R2GH*s, and formed a second distinct group on phylogenetic trees (Fig. 2). The significance of this is examined in detail later.

### Establishing paralogy relationships between *R2GH*s

In order to examine relative orthologous and paralogous relationships among *R2GH*s from the genotypes analyzed, the percentage of nucleotide identity between all pairs of homologs was investigated in the three potato genotypes. *R2GH*s exhibit identities ranging from 77 % to 100 % (87.7 ± 3.2 %). Putative orthologous sequences (identified by positional synteny and phylogenetic analysis) show the highest level of identity ranging from 96.6 % to 100 % (average 99.4 %, 58 pairs exhibit 100 % identity). Paralogs exhibit identities ranging from 69 % to 97 % (average 84.69 %).

Comparisons revealed 4 identifiable sets of highly conserved paralogs exhibiting identities ranging from 92 % to 97 % in the *S. tuberosum* haplotypes (Fig. 1). These highly identical paralogs within individual haplotypes are probably the result of recent duplication events of either single genes or blocks of genes. No pairs of similarly highly conserved paralogs were identified in the tomato *R2GH*s, indicating the absence of duplication events as recent as those in potato.

Identification of potential paralogs on the basis of nucleotide identity further clarified rearrangements between clusters A and B in haplotype HB-H1 (Fig. 1). One pair of *R2GH*s seems to exist in four tandemly arrayed paralogous copies (marked in yellow and orange in Fig. 1) thus the pair is particularly prone to duplication. This paralogous-pair copy is involved in the disruption of colinearity observed between haplotype HB-H1 and the DM/RH/HB orthohaplotype. An additional pair of highly conserved paralogs (marked in blue in Fig. 1) was identified in cluster B in both the DM/RH/HB ortho-haplotype and HB-H1. The insertion from cluster A interrupts the colinearity at cluster B in HB-H1 at this point. The three subsequent *R2GHs* (7, 8 and 9 in Fig. 1) in HB-H1 do not exhibit obvious orthologous relationships with any *R2GH* in the DM/RH/HB orthohaplotype, while orthology is re-established for the last members of cluster B in both haplotypes.

### Two different mechanisms prevail in the evolution of the *R2GH*s

As previously mentioned, two distinct tree topologies can be observed in the dendrogram in Fig. 2 and they correlate with the relative taxonomic relationships associating the *R2GH*s investigated. One pattern essentially corresponds to *R2GH*s from *Solanum* wild species and the other to *R2GH*s from the cultivated *S. tuberosum* genotypes RH, DM and HB. A similar phenomenon has been observed for the *RGC2* disease resistance locus in lettuce, which displays two types of sequence diversity among resistance gene homologs [15]. Kuang et al. proposed that the two different tree topologies in lettuce correspond to two separate types of homologs distinguished by evolutionary rates, the fast evolving Type I *RGH*s and the slow evolving Type II *RGH*s.

In our phylogenetic analysis, RH, DM and HB *R2GH*s exhibit the features of Type II genes: The allelic relationships amongst *R2GH*s can be easily deduced from this tree topology: orthologs form monophyletic clades in the phylogenetic tree with high bootstrap values (generally close to 100 %); branches connecting orthologs are very short (<1 % nucleotide substitution); while branches joining paralogs are very long (>5 % nucleotide substitutions). This behaviour reflects a birth-and-death model of evolution [32].

**Fig. 3:**
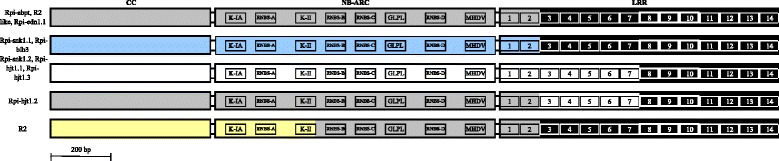
Patterns of sequence identity at the blight resistance functional *R2GH*s. Different colors refer to different gene conversion events. Tracts characterized by the same color and pattern share >99 % identity. The sequences of functional members of the chromosome 4 hotspots for resistance can be summarized into five main combinations of patchworks of sequence similarity. From LRR 8 to LRR 14 the patterns of sequence similarity tend to merge into a unique stretch of sequence identity. Domains and motifs are enclosed in boxes, LRR 8, 9 and 10 are highlighted in bold because their sequence shows 100 % identity level

The tree topology varies for *R2GH*s from *Solanum* wild species: the length of the branches is intermediate (varying between 1 and 6 %) and bootstrap values at the nodes are mostly < 90 %. Hence, this portion of the cladogram assumes the attributes of a Type I resistance gene homolog tree that conceals orthologous relationships among members [10]. Distance analysis also showed that the *R2GH*s known to be actively involved in blight resistance tend to form a monophyletic group also including five non-functional members from *Solanum* wild species *GH2, GH45, GH-D3, GH-8* and *AM-5* (Fig. 2). These R-genes and homologs are mosaics of sequences producing stretches of identity dispersed throughout all domains (Fig. 3). In the blight resistance “functional clade” the LRR domains are highly uniform (black-shaded gene conversion event in Fig. 3), as their products are known to interact, either directly or indirectly with a restricted number of blight effectors [25].

Although phylogenetic analysis supports a slower Type II evolutionary mode for *R2GH*s from DM, RH and HB, this conclusion is complicated by the relative evolutionary distance between different genotypes in the two sections of the tree described above. The strong orthology relationships exhibited by DM, RH and HB *R2GH*s are probably at least partly due to the lower degree of taxonomic separation of these genotypes relative to the wild species. Despite this, the longer branches between paralogs in the DM/RH/HB ortho-haplotype relative to the wild species clade still suggested that the two groups exhibited different modes of evolution. A more definitive footprint of rate of evolution is the extent to which gene conversion events, the major feature of Type I evolution, have homogenised paralogous cluster members, with lower gene conversion rates leading to comparatively longer inter-paralog branches. Thus we expected that these non-reciprocal sequence exchanges, characteristic of Type I evolution would occur at a relatively low rate in the sequences from the DM/RH/HB ortho-haplotype. In order to identify sequence exchanges between *R2GH*s, visual inspection was combined with the output of the program Genconv [33], used to detect possible gene conversion events between all pairs of *R2GH*s. Only five separate sequence exchanges were identified amongst RH, DM and HB *R2* paralogs (Additional file 5) indicating that members of the RH/DM/HB ortho-haplotype have not been subject to frequent exchange of sequences and have evolved mainly through the accumulation of point mutations at hypervariable sites.

This contrasts to what was previously observed for the *R2* gene family members of the *R2* gene family *Solanum* wild species [23, 24]. The same analyses described for the *R2GH*s from RH, DM and HB were performed on *R2GH*s from *Solanum* wild species where we counted a total of 72 gene conversion events. Sequence exchanges were detected amongst functional R-genes and R-gene homologs both at the 5’ and 3’ ends. Surprisingly, they could also be identified amongst members belonging to different *Solanum* wild species. Even if it was not possible to observe chimerism for all homologs, it is unquestionable that sequence exchanges played an important role in diversifying the members of the *Solanum* wild species *R2* gene family (Additional file 5).

Thus both phylogenetic analysis and examination of the evolutionary forces shaping the sequence of *R2GH*s support the existence of two distinct modes of evolution acting in R-gene homologs from different lineages.

## Discussion

### The *R2* hot spot for resistance has an unusually highly conserved multipartite structure in potato and tomato genotypes

One of the most striking features of this analysis is the conserved multipartite structure found at the *R2* locus across potato and tomato genotypes examined in the study, whereby the members of the *R2* gene family are organized into several discrete clusters separated by intervals >50 Kbp coding for structurally and functionally unrelated genes in both species. Three clusters exhibit clear conservation across the potato and tomato genotypes examined in an uninterrupted window of comparison extending approximately 800 kb in potato and 500 kb in tomato. DM possesses an additional adjacent cluster (for which no sequence coverage was available in RH and HB) that is apparently unrepresented in tomato.

The *R2* region has been examined in potato and tomato previously, as part of large scale re-annotations of the R gene complement of both species using capture and re-sequencing approaches [34, 35] In these studies, DM was described as having two clusters in this region, and in the latter, tomato is also described as having two clusters. This differs slightly with the picture we present (up to 4 clusters in potato and 3 in tomato) for two main reasons. In the first instance, we use a slightly different definition of the number of independent non R-gene ORFs required to separate clusters (8 as opposed to 14). This results in a separation of clusters A and B in our study, which are described as a single cluster in both potato and tomato in the previous studies. The main reason we adopted a lower number of intervening genes to define clusters was to highlight the power of the syntenic block of non R gene encoding genes between cluster A and B to clarify the syntenic relationships of clusters between the species and within potato genotypes. In addition, cluster C is not described for potato in either previous study, although, the second, smaller cluster described for tomato by Andolfo et al. [34] does correspond to cluster C. However, the second smaller cluster in potato described in that study is actually the DM specific cluster described in this study. Finer scale delineation of syntenic clusters and inclusion of all syntenic pairs of clusters across both species in this study should bring greater clarity to the organisation of the *R2* region in both species.

Comparison of the *R2* region across potato and tomato in this study allows us to make some broad conclusions regarding the evolutionary history of the locus. The positional conservation of clusters A, B and C, as defined by their flanking regions and similar relative sizes, implies that the existence of these clusters pre-date tomato-potato speciation. The remarkably larger size of cluster A in potato suggests that several duplication events took place in this cluster in potato subsequent to speciation. To examine this, similar to Andolfo et al. [34], we searched for potential duplication events in the potato ortho-haplotype and tomato (although we used straightforward sequence identity comparisons rather than phylogenetic inference, and identified fewer instances of duplication because of the use of a higher threshold to declare putative orthologs). The identification of several sets of highly identical paralogs in cluster A clearly supports multiple duplication events in the potato lineage, and at least one duplication event is also evident in cluster B.

The DM specific *R2GH* cluster lacks a syntenic cluster in tomato. Given the history of duplication in potato, it is perhaps more likely that this cluster arose in potato after speciation, but the converse (loss in the tomato lineage) cannot be excluded. The lack of highly identical paralogs within the clusters in tomato cv. Heinz 1703 using a threshold at which they are identifiable in the potato lineage indicates the absence of recent duplication events in this tomato cultivar, perhaps suggesting that the organisation of the cluster in tomato is more similar to that of the ‘pre-speciation’ structure of the locus. While the inter cluster sequences exhibit clear orthology, only three *R2GH*s showed a high level of conservation between potato and tomato (possibly indicating a selective force such as a common pathogen, acting across species boundaries), but no clear orthology exists between remaining homologs when compared across these species.

### Patterns of evolution in the R-gene family in *S. tuberosum* genotypes

We characterised the *R2* region in three *S. tuberosum* genotypes with a large contribution from cultivar Group Phureja in their background. Comparison of our data to previously available data from allele mining studies in blight resistant wild potato species indicates the acquisition of two distinct patterns of evolution by different lineages, based on both phylogenetic and gene conversion analysis.

Phylogenetic distance analysis tends to keep RH, DM and HB homologs separated from wild species members and reveals two different tree topologies for the respective groups. Branches connecting RH, DM and HB *R2GHs* are either very short or very long, while branches connecting *R2GH*s from *Solanum* wild species have mostly medium lengths. This distinction into clades in which paralogs/orthologs can be clearly defined (DM/RH/HB) or in which paralogy relationships are obscured (wild species) has previously been shown to be indicative of the acquisition of differing rates of evolution by members of the same R-gene family at a single locus [19].

The evolutionary mechanism underlying the above distinction was investigated using gene conversion analysis, and it was apparent that non-reciprocal sequence exchange was a low frequency event between paralogs in the DM/RH/HB ortho-haplotype, a feature diagnostic of Type II R-genes. The low level of identity between paralogs conceals their evolutionary relationships, with the exception of four copies of highly identical paralogs that are expected to be the result of recent duplication events. This mode of evolution supports a birth and death process that starts with duplication, followed by diversification, neofunctionalization, or silencing of the original genes to generate new distinct paralogs [36]. On the other hand, *Solanum* wild species homologs are chimaeric sequences, exhibiting the footprint of extensive non-reciprocal sequence exchanges characteristic of the fast evolution rates typical of Type I R-genes.

The apparent difference between type I and type *II R2GH*s examined begs the question as to the source of these distinct evolutionary modes. The taxonomic separation of the two sets of species examined is related to their centres of origin: the *Solanum* wild species examined have all Mexican origins, while the *S. tuberosum* genotypes have South American origins [37]. It seems a reasonable hypothesis that the different geographic distributions have led to different evolutionary pressures, the most obvious difference being the fact that the Mexican species have evolved in the geographic centre of diversity of the most devastating pathogen of potato, *P. infestans*. The necessity to adapt and evolve new R-gene specificities in the centre of diversity of the late blight pathogen has apparently fundamentally shaped the evolutionary dynamics of the *R2* locus in the Mexican species, while the lack of this pressure has resulted in a more gradual pace of diversification in the South American derived species. The conserved evolution amongst members of the *R2* gene family from the tuber bearing potato and the fruit tomato contrasts with the rapid selection prevailing in *Solanum* wild species, whose diversification unquestionably occurred after tomato/potato speciation.

It is worth pointing out that there are some limitations to the hypothesis advanced above based on our data. As mentioned above, the diversity of the genotypes we examined is relatively narrow and probably largely reflects *S. tuberosum*. Thus, although we can definitively say that *R2GH*s from this specific South American background have experienced the slower Type II evolutionary mode, we hesitate to extend this to other species of similar geographic origin. In addition, the manner in which the wild species *R2GH*s were isolated, through a process of allele mining, has possibly introduced a bias towards genes that are structurally similar to functional members initially identified by map based cloning, and we also lack the contextual information for the wild species provided by BAC and WGS sequencing in the *S. tuberosum* genotypes. So, while we can reasonably posit that the faster, Type I mode of evolution is occurring in wild species, we cannot exclude the simultaneous occurrence of Type II evolution at the *R2* locus in these genotypes. Indeed, the occurrence of Type I and Type II evolution in the same R-gene family has previously been observed [19].

## Conclusions

**Fig. 4:**
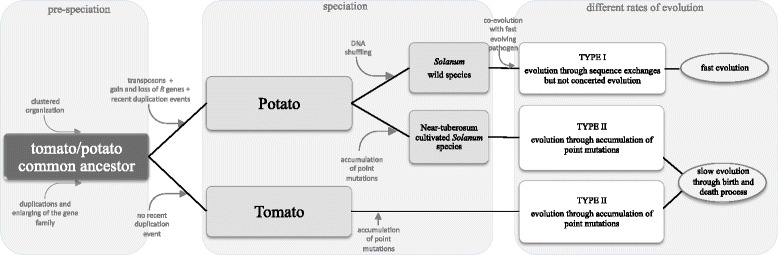
Flowchart of the proposed evolutionary model of members of the R2 gene family

Comparisons between R genes from the *R2* locus in tomato and cultivated and wild potato have allowed us to describe the divergent evolutionary path of the locus in these lineages (Fig. 4). The *S. tuberosum* genotypes investigated in this study have maintained a multipartite clustered structure at the *R2* locus that predates speciation from tomato. *R2GH*s from DM, RH, HB and tomato have evolved mostly through a birth and death process, where duplications, accumulation of point mutations and transposition events have brought about the current organization of the *R2* region. On the other hand, gene conversions played a major role in shaping the evolution of R-gene homologs from *Solanum* wild species. Despite the fact that R-gene loci can encode multiple disease resistance specificities, it is possible that in its centre of diversity, a single aggressive pathogen can act as the major driving force to shape the evolutionary process at any such locus. In the absence of this strong selective pressure the same R-gene locus may follow a very different path in different lineages.

## Methods

### Sequences used in this study

Potato sequences from the DM1-3 516R44 genotype (PGSC_DM_v3_2.1.10_pseudomolecule_AGP(1)) were downloaded from the Potato Genome Sequencing Consortium Public Data Release (solanaceae.plantbiology.msu.edu/pgsc_download.shtml). The BAC end sequences (BES) of the RHPOTKEY library were downloaded from the GSS division of Genbank. Hein et al. [38] provided the BAC end sequences of HB BAC clones containing copies of *R2GH*s. Tomato cv. Heinz 1703 (HZ) whole genome sequences (Tomato WGS scaffolds, SC2.40) were downloaded from the *Solanum* Genomics Network (https://solgenomics.net).

Potato BAC sequences were generated as described below. R-gene sequences *GHA, Rpi-abpt*, *GH10, GH11, GH12, GH13, GH14, GH15, GH16, GH2, GH3, GH45, GH5, GH7, GH8, GH9, GHD3, R2, AM2*, *AM3, AM5, ednGH3*, *ednGH4*, *ednGH5*, *ednGH6*, *ednGH7*, *ednGH8*, *R2-like, Rpi-edn1.1*, *hjtGH1*, *Rpi-hjt1.1*, *Rpi-hjt1.2*, *Rpi-hjt1.3*, *Rpi-snk1.1*, *Rpi-snk1.2*, *snkGH2*, *snkGH5*, *snkGH6*, *snkGH7*, *Rpi-blb3* and *SBA* [8, 9] were downloaded from Genbank (accessions FJ536324-FJ536346; GU563963- GU563979).

Identification and sequencing of potato BAC sequences spanning the *R2* region.

The identification and sequencing of the BAC clone GB003E01 from the GB BAC library (built on the RH89-039-16 genotype, 35,700 clones, average insert size 102 Kbp) are described in Moloney et al. [39]. BAC clones RH049C05, RH089H21, RH082P19, RH103C24, RH131E21, RH005L24, RH175J12 and RH107O01 from the RHPOTKEY BAC library (generated from the RH89-039-16 genotype, 78,000 clones, average insert size 120 Kbp) were identified through a combination of chromosome walking from the BAC clone GB003E01 and alignment the RHPOTKEY BES to the sequence of the tomato genomic region syntenic to the *R2* locus. The HB BAC clones HB036I05, HB040O06, HB016P11 and HB001G02 from the HB171(13) BAC library (280,000 clones, average insert size 100 Kbp) were identified aligning the HB BES obtained as described in Hein et al. [38], against the sequence of the RH BAC clones. Similarly, the sequence of the RH BAC clones aided identifying DM superscaffolds.

### BAC sequencing and assembly

Twelve BACs from RH and HB were sequenced for this study. The BAC clones were sequenced to a minimum six-fold coverage using Sanger sequencing methods on ABI 3730 xl by the company GATC, Germany. The BAC sequences were assembled using the assembler program Gap4 from the Staden Package (MRC Rosalind Franklin Centre for Genomics Research, Cambridge, UK) to a minimum standard of HTGS Phase II. All sequences were previously trimmed to remove sequencing vector, cloning vector and *Escherichia coli* contaminant sequences. The automated assembly was brought from HTGS phase I to HTGS Phase II/III by aligning the contigs to overlapping BAC sequences or to the tomato orthologous BAC sequences. Gaps between contigs were filled by exploiting sequences from overlapping BAC clones or by PCR. BACs were submitted to Genbank under the following accession numbers: KM502302 (RH103C24), KM502303 (HB016P11), KM502304 (HB011I06), KM502305 (HB015I19/HB036I05), KM502306 (HB026A19), KM502308 (RH131E21), KM502309 (HB040O06), KM502310 (RH082P19), KM502311 (RH005L24), KM502312 (HB001G02), KM502313 (RH175J12), KM502314 (RH107O01). An additional three BACs, previously submitted to GenBank as AC233622 (RH089H21), AC233613 (RH049C05) and AC236731 (GB003E01) were also advanced from HTGS Phase I to Phase II/III, as described above, for the study.

### Nomenclature of the R-gene sequences

The R-genes sequences from RH, DM and HB and tomato cultivar Heinz were named to distinguish among genotypes, R-gene cluster of affiliation and phases. The first 2 letters indicate the genotypes (RH, DM, HB, HZ) followed by a number (0 or 1) that designates the phases. The letters A, B or C, reflect the cluster of affiliation. The last three numbers distinguish individual R-gene sequences.

### Sequence alignments, phylogenetic and diversifying selection analysis

DNA alignments were performed using ClustalW 2.0.12 [40] keeping default settings. The alignments were manually redefined using Jalview [41], and afterwards, used to create neighbour-joining trees using Kimura’s two parameter model. The bootstrap values were calculated using PAUP*4.0. (Sinauer Associates, Sunderland, MA). Phylogenetic trees were displayed using the program Figtree v.1.3.1.

Sequence exchanges were detected either visually or using the statistical program for detecting gene conversions GENECONV version 1.18 [33]. GENECONV was run using default settings. Recombinations were considered to be present in the exchanged tracts if the global *P* value was <0.05. Each event was visually confirmed and when a gene showed exchanged sequences with multiple homologs at the same position, only the longest conversion tract was taken into account. Separated events between two sequences were joined into a longer conversion tract when separated by just one mismatch.

## Additional files

Additional file 1:
**Physical map of the R2 region combining the location of genetic markers anchored to the region, to the location of genomic sequences and R-genes.** The physical location of the sequence of genetic markers used to map quantitative and qualitative resistance at the R2 region is represented on the top of the figure (STM3016 from Milbourne et al. [42]; T1430, C2_At5g04810, TG123, TG370_F, T437_R, cLPT5-B19 from Bombarely et al.[43]; Th21 from Park et al. [44], C237 from Moloney et al.[39]; 11_4_f, 107O01_52, 40SSR2_f from Destefanis et al. unpublished results). Three potato genotypes (HB, DM and RH from the top to the bottom) are separated from the orthologous tomato region by the measuring scale. BAC and scaffold sequences are shown as solid lines, gaps are displayed as dotted lines. R2GHs are represented as filled boxes of assigned orientation. Red boxes represent full length open reading frames; black boxes designate analyzed pseudogenes, while grey boxes are pseudogenes not included in the analysis. The dotplot was obtained from the alignment of the *R2* sequence (accession FJ536325) and the potato or tomato BAC/scaffold sequences. Putative orthologous pairs of *R2GH*s are inter-connected by dotted lines. Transposable elements are represented on the sequences as triangles of assigned orientation. Light gray-shaded areas cover three R-gene clusters, the proximal cluster C, followed by cluster B and the distal cluster A. The small cluster specific to DM, for which there is no apparent orthologous cluster in tomato (and for which we have no coverage in RH and HB) is illustrated on the right. The figure is drawn to scale. (PDF 197 kb) Additional file 2:
**Gene annotation of the** ***R2*** **locus in potato (** ***S. tuberosum*** genotypes RH089-039-16, HB171(13), DM1-3 516R44) and tomato (*S. lycopersicum* cv. Heinz 1706). For each scaffold/BAC clone are indicated: linkage phase, genotype of affiliation, GenBank accession (where available), description of the product and EST accession (from the SolEST database). Manual annotation of DM0-3 516R44 and Tomato cv. Heinz were compared to the automated annotation and no relevant differences were identified. (XLSX 27 kb) Additional file 3:
**Microsyntenic blocks between potato (RH-H0 haplotype) and tomato annotations.** Microsyntenic proteins (same function, location and orientation) are lined up in the same row. Proteins shaded in grey are included in resistance clusters and cannot be directly compared because of fractioned synteny [45]. Putative transposable elements are shaded in red. Microsyintenic blocks are framed. (XLSX 15 kb) Additional file 4:
**Summary table of the number of** ***R*** **gene homologues identified at the** ***R2*** **region.** (XLSX 9 kb) Additional file 5: Gene conversion events identified among *R2GH*s in RH, DM and HB, and among *R2GH*s in *Solanum* wild species. Gene conversions were visually identified or using the program Genconv [33]. A number was attributed to each event. For each gene conversion event are indicated details on the involved sequence pairs, position, size and ρ value. (XLSX 360 kb)

## Abbreviations

BAC: Bacterial artificial chromosome
Kbp: Kilobase pair
LRR: Leucine rich repeat
NB: Nucleotide binding
ORF: Open reading frame
QRL: Quantitative resistance locus
QTL: Quantitative trait locus
R-gene: Disease resistance gene
RGH: Resistance gene homolog
R2GH: *R2* Resistance gene homolog
UTR: Untranslated Region
WGS: Whole Genome Sequencing
