# Supplementary figures and images for: A disease resistance locus on potato and tomato chromosome 4 exhibits a conserved multipartite structure displaying different rates of evolution in different lineages
Source: BMC Plant Biol. 2015 Oct 24;15:255. doi: 10.1186/s12870-015-0645-8 (PMC4619397; doi:10.1186/s12870-015-0645-8)

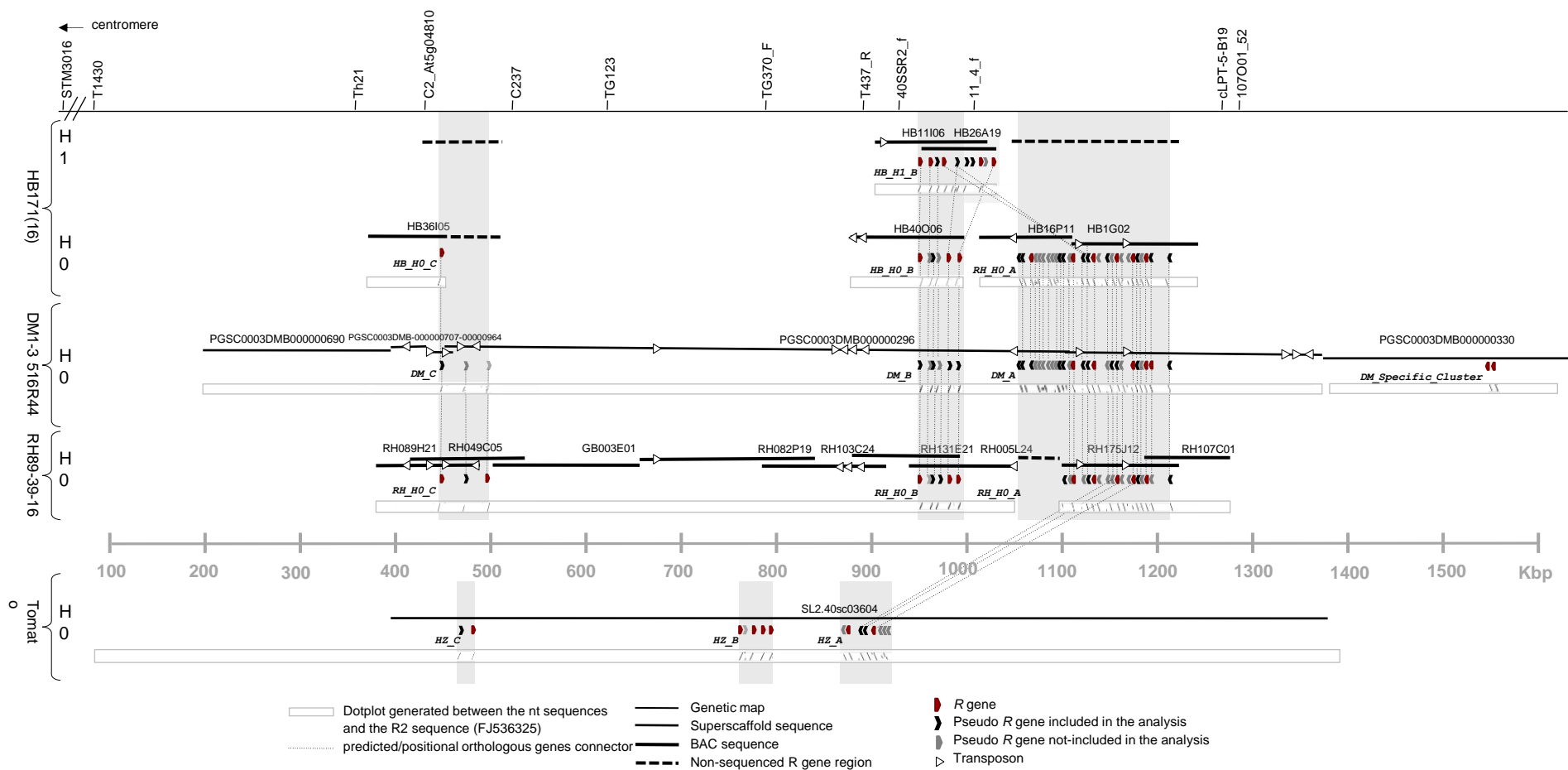

Supplement: Additional file 1: — Physical map of the R2 region combining the location of genetic markers anchored to the region, to the location of genomic sequences and R-genes. The physical location of the sequence of genetic markers used to map quantitative and qualitative resistance at the R2 region is represented on the top of the figure (STM3016 from Milbourne et al. [42]; T1430, C2_At5g04810, TG123, TG370_F, T437_R, cLPT5-B19 from Bombarely et al.[43]; Th21 from Park et al. [44], C237 from Moloney et al.[39]; 11_4_f, 107O01_52, 40SSR2_f from Destefanis et al. unpublished results). Three potato genotypes (HB, DM and RH from the top to the bottom) are separated from the orthologous tomato region by the measuring scale. BAC and scaffold sequences are shown as solid lines, gaps are displayed as dotted lines. R2GHs are represented as filled boxes of assigned orientation. Red boxes represent full length open reading frames; black boxes designate analyzed pseudogenes, while grey boxes are pseudogenes not included in the analysis. The dotplot was obtained from the alignment of the R2 sequence (accession FJ536325) and the potato or tomato BAC/scaffold sequences. Putative orthologous pairs of R2GHs are inter-connected by dotted lines. Transposable elements are represented on the sequences as triangles of assigned orientation. Light gray-shaded areas cover three R-gene clusters, the proximal cluster C, followed by cluster B and the distal cluster A. The small cluster specific to DM, for which there is no apparent orthologous cluster in tomato (and for which we have no coverage in RH and HB) is illustrated on the right. The figure is drawn to scale. (PDF 197 kb) [file 12870_2015_645_MOESM1_ESM.pdf]
